# Supplementary material for: Changes in global groundwater organic carbon driven by climate change and urbanization
Source: Nat Commun. 2020 Mar 9;11:1279. doi: 10.1038/s41467-020-14946-1 (PMC7062877; doi:10.1038/s41467-020-14946-1)
Supplement: Supplementary file 1 — Supplementary Information [file 41467_2020_14946_MOESM1_ESM.pdf]

Supplementary Information for:

Changes in global groundwater organic carbon driven by climate change and urbanization

by

McDonough et al.

Correspondence to: lizam@ansto.gov.au

Supplementary Figures

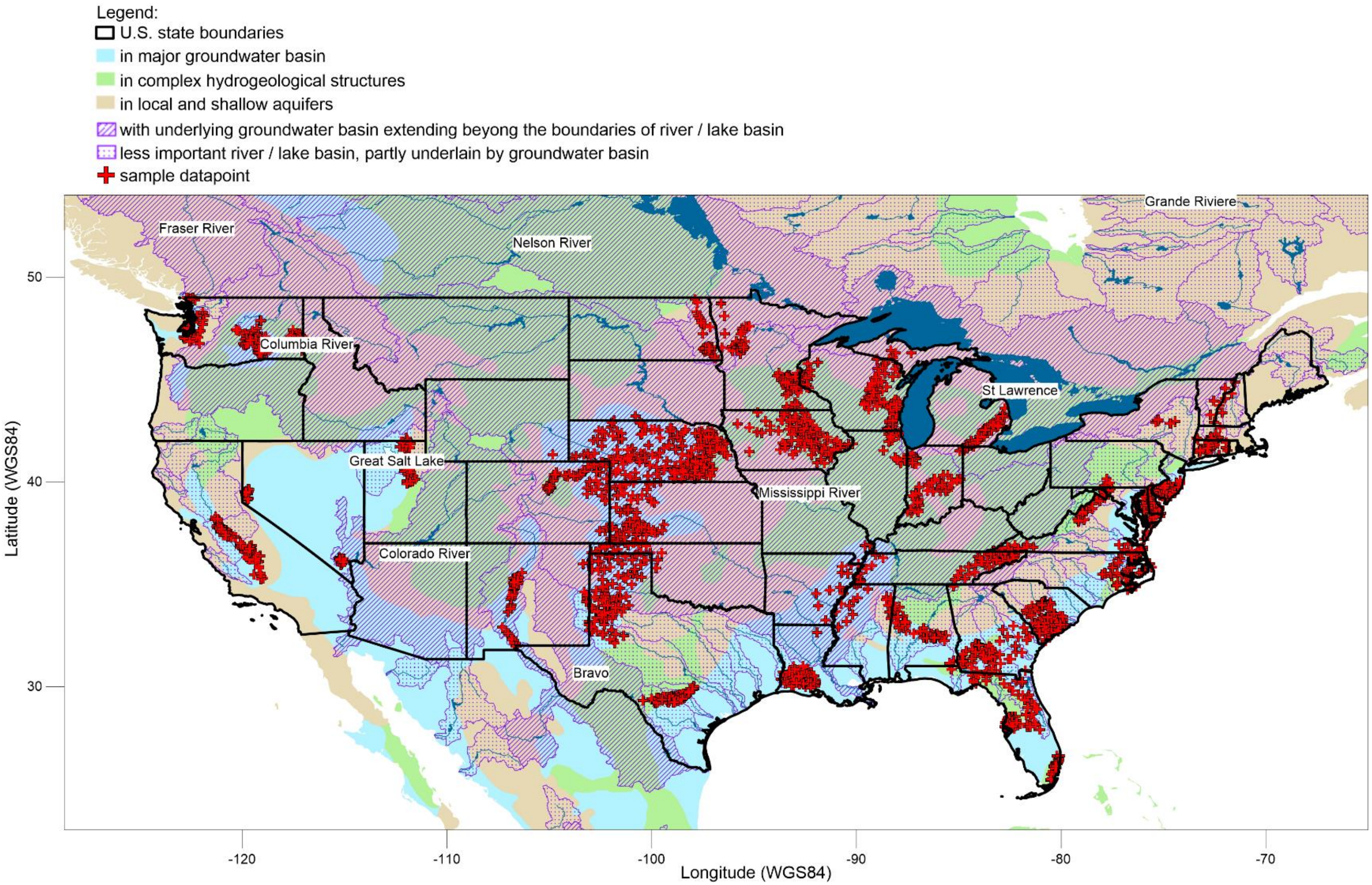

Supplementary Figure 1

US NWQMC data (red plus symbols) [1] overlain over global aquifer WHYMAP data [2].

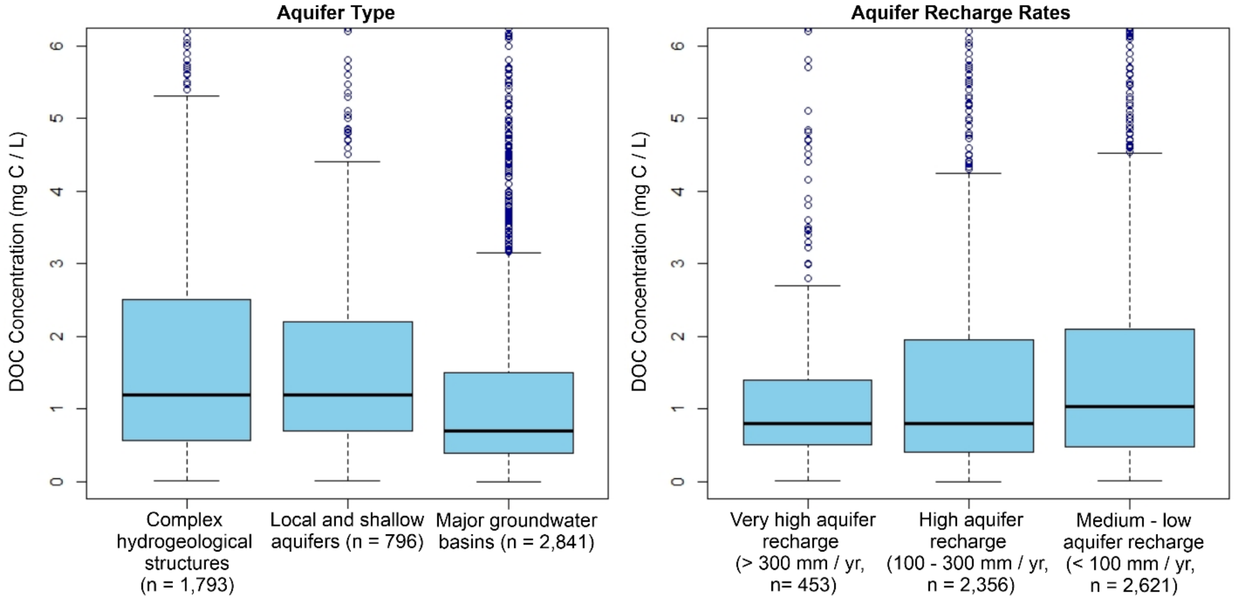

Supplementary Figure 2

Comparison of groundwater DOC concentrations between aquifer type and recharge rates for US NWQMC data using global aquifer WHYMAP data [2]. Boxes represent the interquartile range, containing median, with whiskers representing the 1.5 times the interquartile range of data. Datapoints beyond this are shown as outliers (dark blue circles). Median DOC concentrations in major groundwater basins were significantly lower than in complex hydrogeological structures or local and shallow aquifers (both $p < 2.2 \times 10^{-16}$). Groundwater DOC concentrations in aquifers with medium – low recharge rates (< 100 mm year^{-1}) were significantly higher than in aquifers with 100 - 300 mm year^{-1} and > 300 mm year^{-1} ($p = 2.342 \times 10^{-7}$ and 4.857×10^{-5} respectively). Outliers greater than 6 mg L^{-1} have been removed for clarity (n = 154 [complex hydrogeological structures], n = 26 [local and shallow aquifers], n = 107 [major groundwater basins], n = 15 [very high aquifer recharge (> 300 mm year^{-1})], n = 153 [high aquifer recharge (100-300 mm year^{-1})] and n = 119 [medium – low aquifer recharge (< 100 mm yr^{-1})].

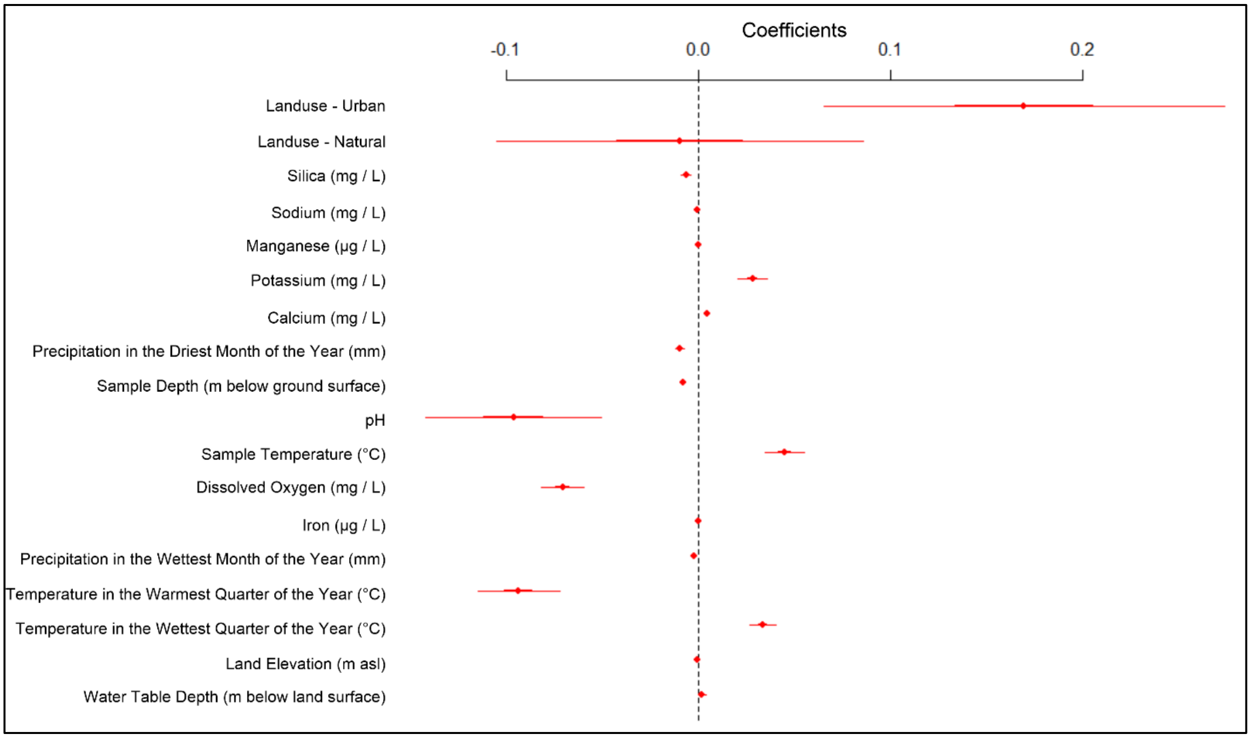

Supplementary Figure 3

Regression estimates of the effects of model variables on groundwater DOC concentrations. Centre points represent mean regression estimates with inner (thicker) bars representing 50% confidence intervals and outer (thinner) bars representing 95% confidence intervals. Regression estimates from top to bottom are 0.17, $-9.74x10^{-3}$, $-6.29x10^{-3}$, $-5.66x10^{-4}$, $3.75x10^{-5}$, $2.83x10^{-2}$, $4.45x10^{-3}$, $-9.53x10^{-3}$, $-7.77x10^{-3}$, $-9.61x10^{-2}$, $4.49\ x10^{-2}$, $-7.06x10^{-2}$, $6.87x10^{-5}$, $-2.45x10^{-3}$, $-9.33x10^{-2}$, $3.35x10^{-2}$, $-2.66x10^{-4}$ and $1.84x10^{-3}$ (also listed in Supplementary Table 2).

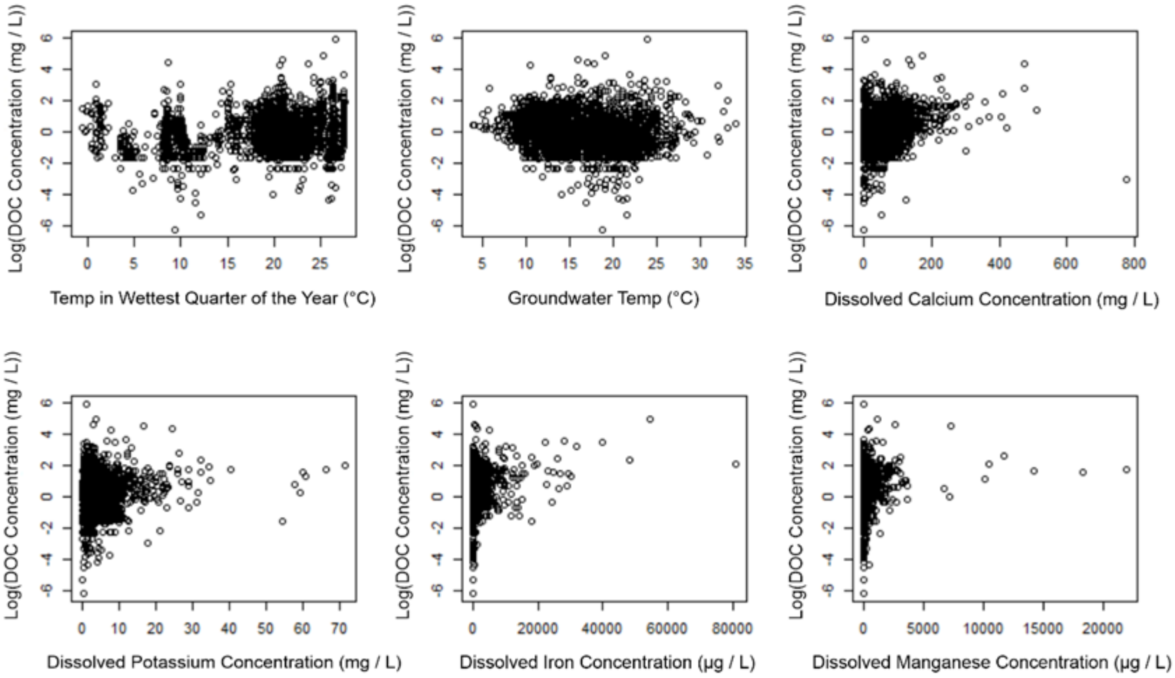

Supplementary Figure 4

Simple scatterplots of model variables with positive correlations ($p < 0.05$) with log(*DOC concentration (mg / L)*). NB: these plots show only the correlation between log(DOC concentration*(mg / L)*) and individual variables. They do not account for the other variables included in the model and do not represent model results.

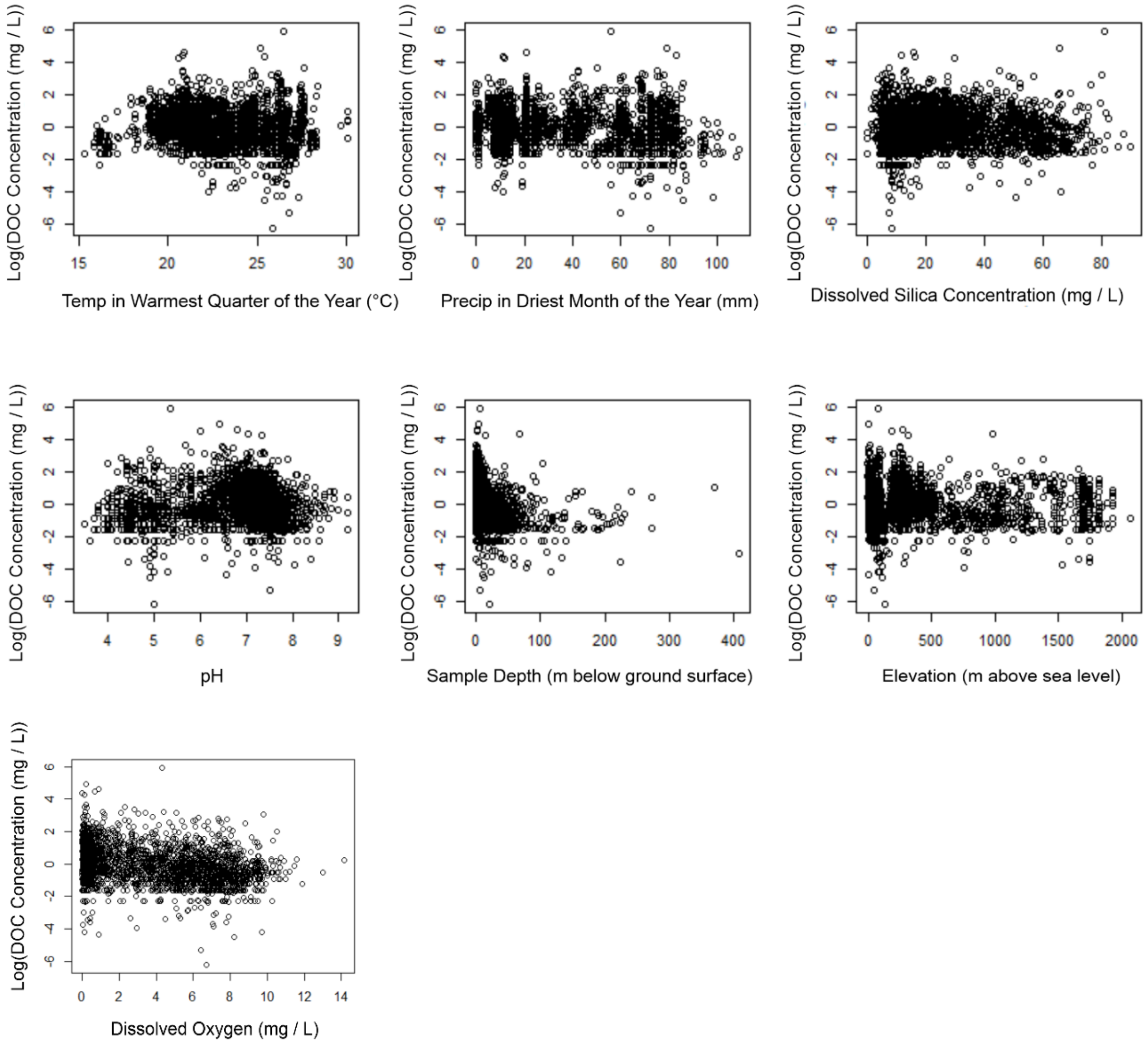

Supplementary Figure 5

Simple scatterplots of model variables with negative correlations ($p < 0.05$) with log(*DOC concentration (mg / L)*). NB: these plots show only the correlation between log(DOC concentration*(mg / L)*) and individual variables. They do not account for the other variables included in the model and do not represent model results.

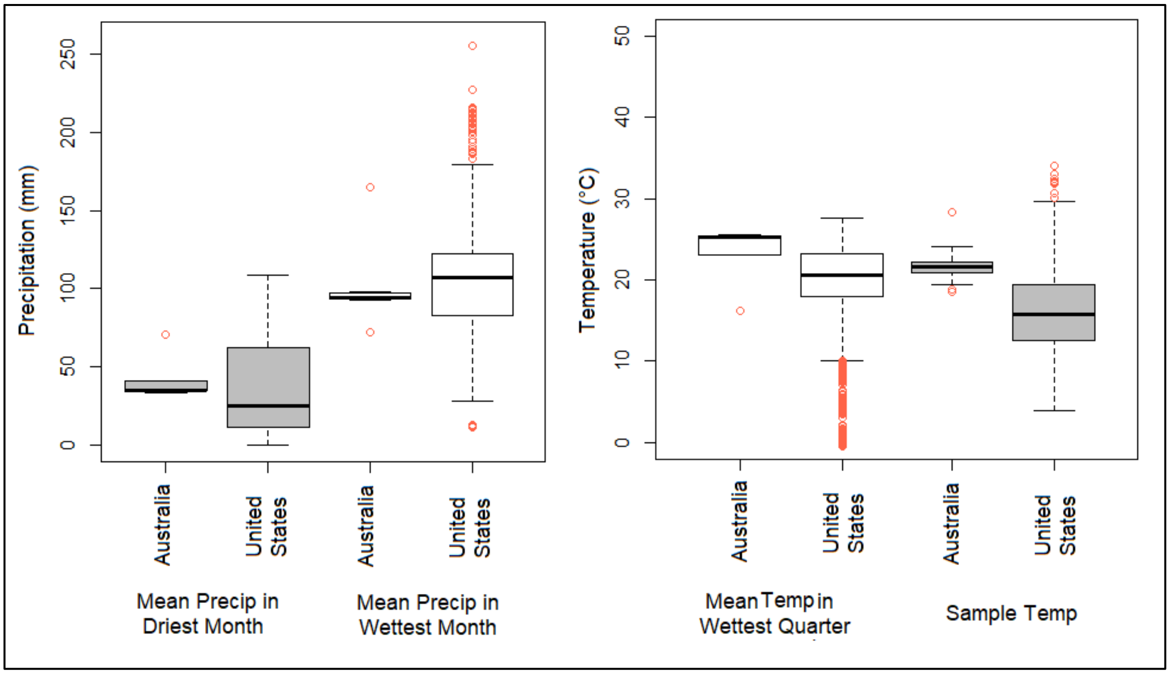

Supplementary Figure 6

Boxplots comparing variables which show opposing correlations in the Australian and US datasets. Boxes represent the interquartile range, containing median, with whiskers representing the 1.5 times the interquartile range of data. Datapoints beyond this are shown as outliers (red circles).

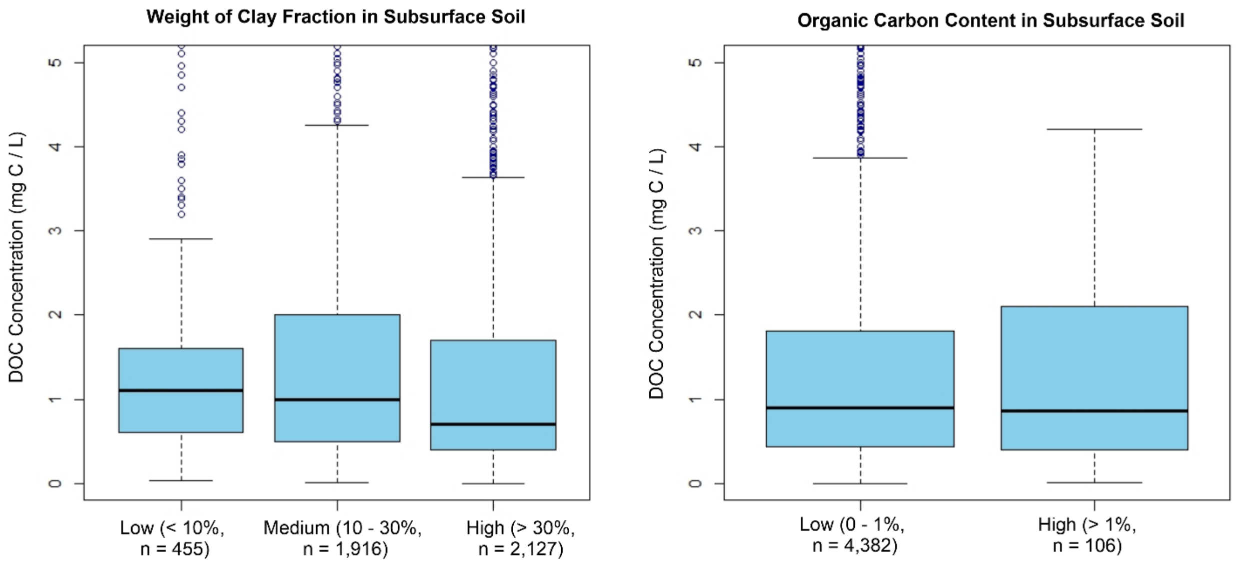

Supplementary Figure 7

Comparison of groundwater DOC concentrations with varying subsurface percent weight of clay and organic matter content using Regridded Harmonized World Soil Database v1.2 [3]. Boxes represent the interquartile range, containing median, with whiskers representing the 1.5 times the interquartile range of data. Datapoints beyond this are shown as outliers (dark blue circles). Median DOC concentrations in soils with high clay percent weight (> 30%) are significantly lower than areas where the subsoil clay percent weight is medium (10 – 30%, $p = 5.628 \times 10^{-13}$) or low (< 10%, $p = 5.533 \times 10^{-8}$). Groundwater DOC concentrations in aquifers with low (0 – 1 %) and high (> 1 %) soil organic carbon content are not significantly different ($p = 0.4723$). Outliers greater than 5 mg / L have been removed for clarity (n = 25 [low (< 10%) clay fraction in subsoil], n = 108 [medium (10 – 30 %) clay fraction in subsoil], n = 124 [high (> 30%) clay fraction in subsoil], n = 253 [low (0 – 1%) organic carbon content in subsoil], and n = 4 [high (> 1%) organic carbon content in subsoil]. N.B. this data represents agricultural and natural areas only due to the potential for paved urban areas to affect infiltration of DOC through the subsoil.

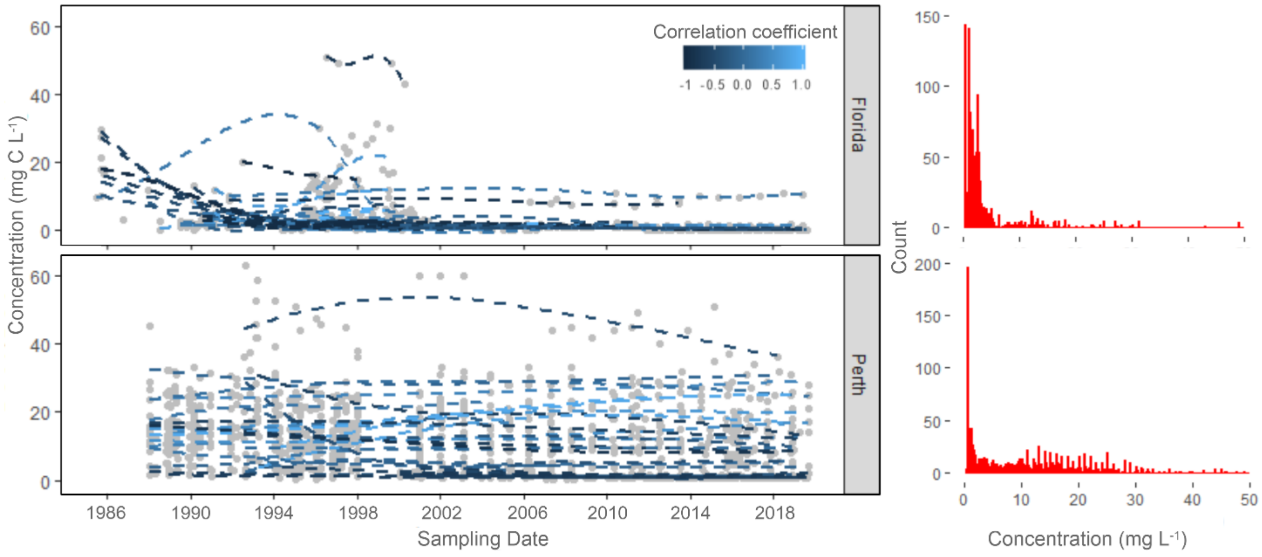

Supplementary Figure 8

Timeseries of groundwater TOC concentrations in south-west Florida, United States (upper left plot) with corresponding histogram shown on the upper right. Timeseries of groundwater DOC concentration data in Perth, Australia (lower plot) with corresponding histogram shown on the lower right). TOC data used for Florida due to the paucity of groundwater DOC datasets available. Here we assume that majority of the TOC in groundwater is dissolved. Both datasets represent currently residential areas. Grey dots represent individual concentration data with dashed lines representing locally estimated scatterplot smoothing (LOESS) colored by correlation coefficient for individual bores (n = 45 bores and n = 51 bores for Perth and Florida, respectively). LOESS smoothing used as many datasets are non-linear. The data suggests a mix of trends including increasing concentrations, decreasing concentrations and no change in concentrations over time. Florida and Perth data were provided by the Southwest Florida Water Management District and Water Corporation (Western Australia) respectively.

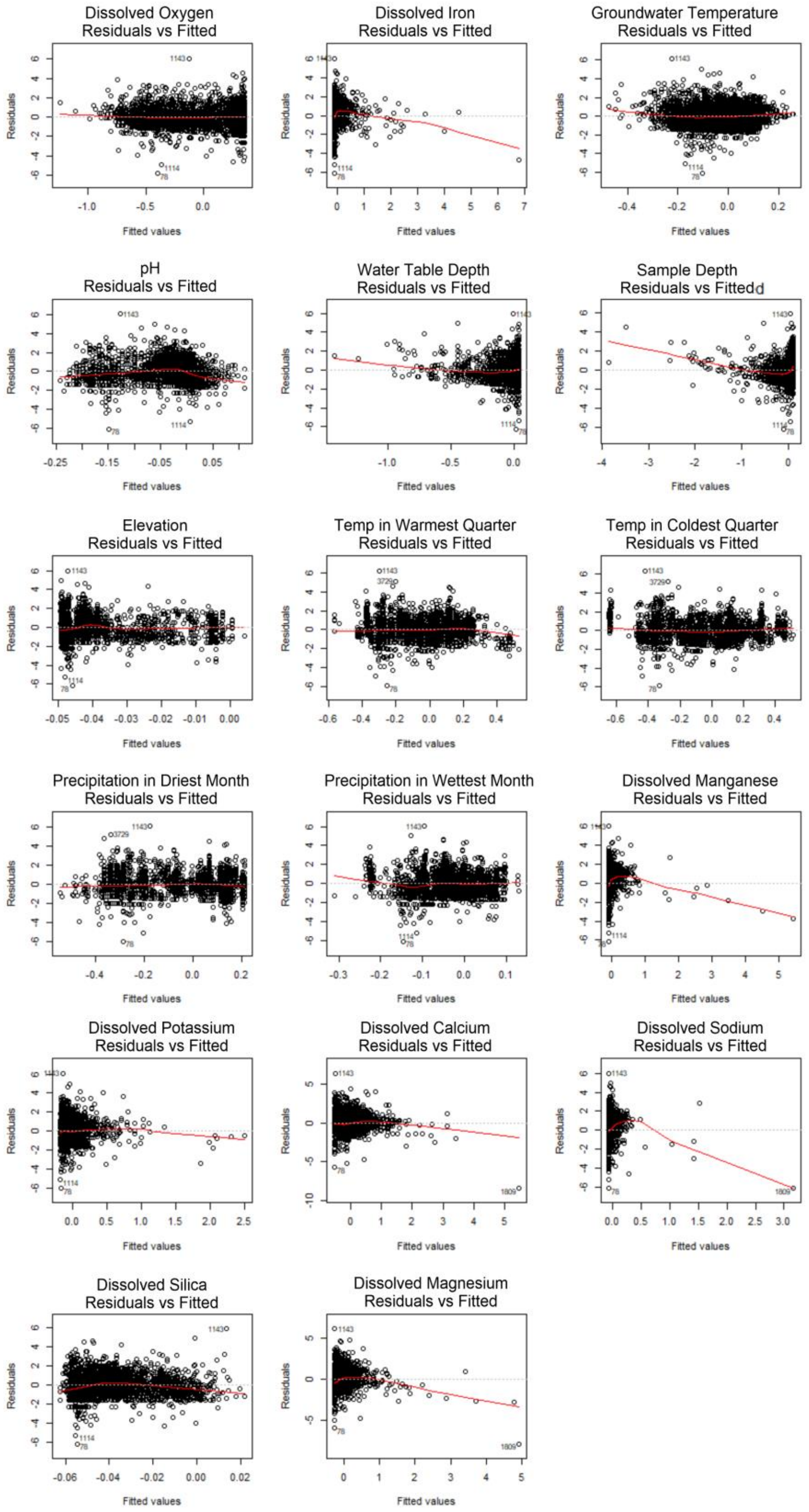

Supplementary Figure 9

Residuals vs Fitted plots for all quantitative variables used in the model.

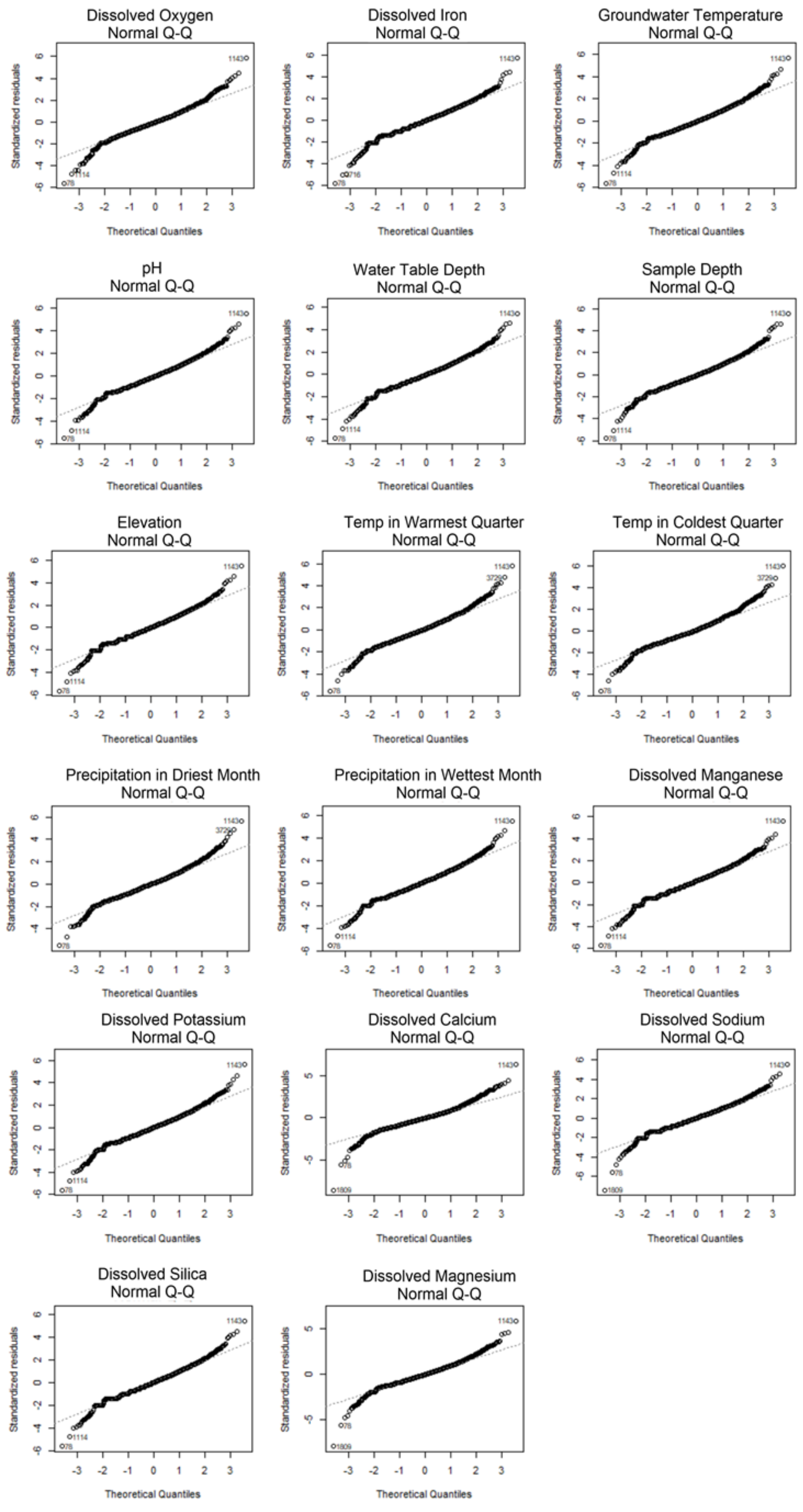

Supplementary Figure 10

Q-Q plots for all quantitative variables used in the model.

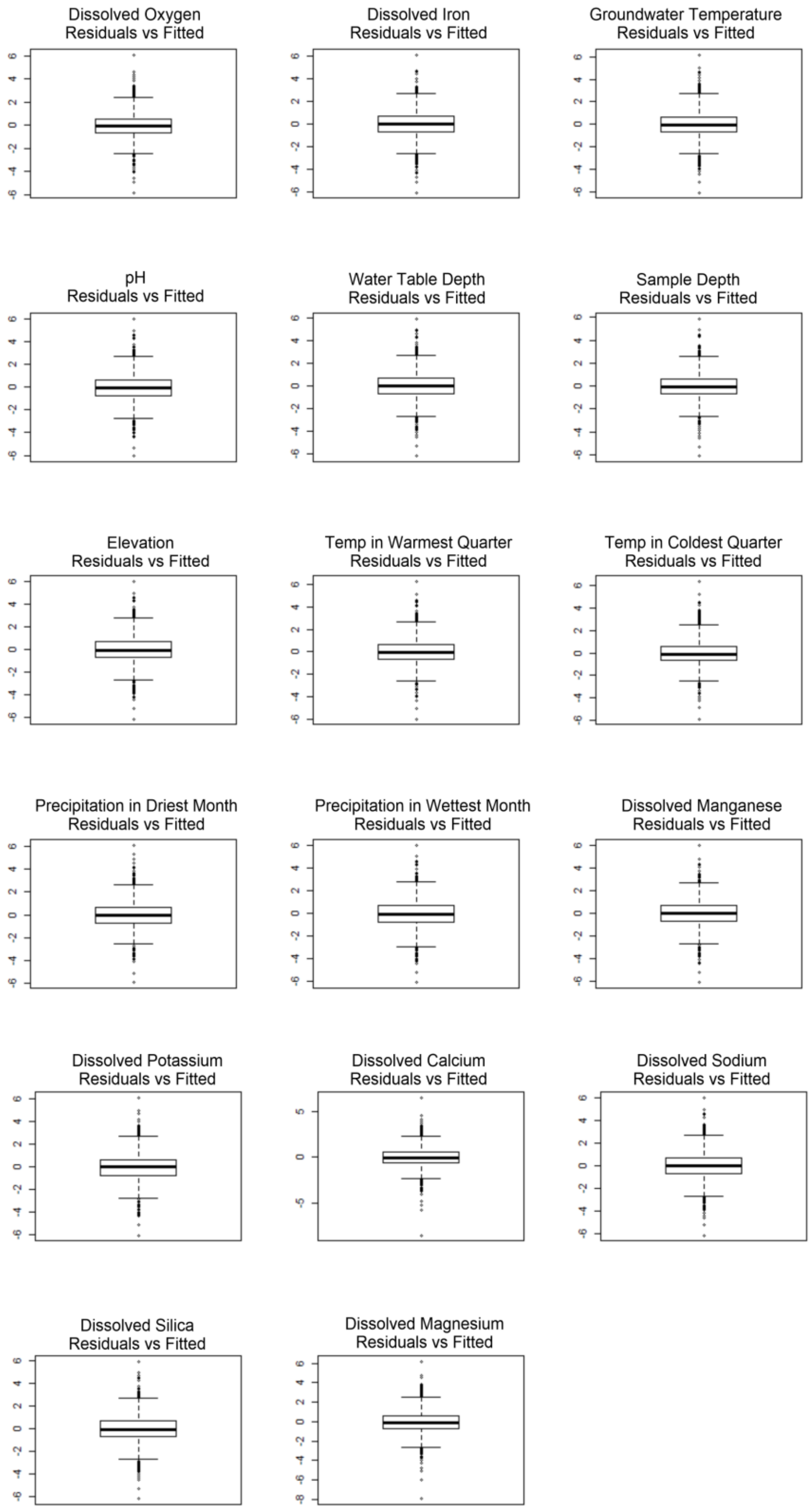

Supplementary Figure 11

Box plots of residuals for all quantitative variables used in the model. Boxes represent the interquartile range, containing median, with whiskers representing the upper and lower 25% of data. The circles shown outside of the whiskers represent outliers.

Supplementary Tables

Supplementary Table 1

Summary of compiled data for groundwater DOC concentration comparison between countries.

Source	Country	n	Data location	DOC filtration size (µm)
4	Algeria	5	Data provided by authors of published source	0.45
5	Australia	29	https://www.sciencedirect.com/science/article/pii/S0048969717314961?via%3Dihub#t0005	0.2
6		8	Data provided by authors of published source	0.7
7		10	Data provided by authors of published source	0.7
8		15	Data provided by authors of published source	0.7
9		17	Data provided by authors of published source	0.45
10		33	Data provided by authors of published source	0.45
11		2	Data provided by authors of published source	0.45
12		5	Data provided by authors of published source	0.7
13		74	Data provided by authors of published source	0.7
14		10	Data provided by authors of published source	0.7
15		14	Data provided by authors of published source	0.7
16		10	Data provided by authors of published source	0.7
17		38	Data provided by authors of published source	0.7
18, 19, 20, 21, 22, 23, 24, 25, 26, 27, 28, 29, 30, 31, 32, 33		1,909	Data provided by co-authors to this paper, Water NSW and Water Corporation W.A.	0.45
34	Argentina	15	Data provided by co-authors to this paper	0.7
35	Bangladesh	13	Data provided by co-authors to this paper	0.45
34	Brazil	30	Data provided by co-authors to this paper	0.7
36	China	17	https://www.sciencedirect.com/science/article/pii/S0048969715301091#t0005	0.45
37		17	www.swdzgcdz.com/oa/pdfdow.aspx?Sid=201303	0.45
31	Cook Island	17	Data provided by co-authors to this paper	0.7
38	France	4	https://www.sciencedirect.com/science/article/pii/S004896970400155X?via%3Dihub	0.45
39	Germany	34	https://www.sciencedirect.com/science/article/pii/S0883292799000219	0.45
40	Canada	52	Data provided by authors of published source	0.45
41		3	https://agupubs.onlinelibrary.wiley.com/doi/epdf/10.1029/WR026i012p02949	0.45
42		12	https://www.sciencedirect.com/science/article/pii/016896229190032R	0.45
43	Belgium	18	Data provided by authors of published source	0.45
	Czech Republic	104	Data provided by authors of published source	0.45

	Denmark	20	Data provided by authors of published source	0.45
	Estonia	19	Data provided by authors of published source	0.45
	France	7	Data provided by authors of published source	0.45
	Malta	8	Data provided by authors of published source	0.45
	Poland	40	Data provided by authors of published source	0.45
	Portugal	28	Data provided by authors of published source	0.45
	Spain	10	Data provided by authors of published source	0.45
	United Kingdom	113	Data provided by authors of published source	0.45
44	Ethiopia	44	Data provided by co-authors to this paper	0.45
45	Iceland	24	Data provided by co-authors to this paper	0.45
35	India	79	Data provided by authors of published source	0.45
46	Kenya	36	Data provided by co-authors to this paper	0.45
47	Mali	12	Data provided by co-authors to this paper	0.45
48, 49	Malawi	41	Data provided by co-authors to this paper	0.45
		48	Data provided by co-authors to this paper	0.45
50	Nepal	40	Data provided by co-authors to this paper	0.45
51	Nigeria	35	Data provided by co-authors to this paper	0.45
52	Scotland	270	Data provided by authors of published source	0.45
53	Senegal	22	Data provided by co-authors to this paper	0.45
54, 55	Uganda	20	Data provided by co-authors to this paper	0.45
		51	Data provided by co-authors to this paper	0.45
56	United States	9	https://esajournals.onlinelibrary.wiley.com/doi/10.1890/0012-9658%282000%29081%5B3133%3AOCSAMI%5D2.0.CO%3B2	0.22
40		5459 [1]	Data provided by authors of published source	0.45
57		99	https://pubs.usgs.gov/journal/1974/vol2issue3/report.pdf	0.45
58		89	Data provided by authors of published source	0.7
59		156	Data provided by authors of published source	0.7
60	Zambia	110	Data provided by authors of published source	0.45
Notes	(1) Sample number after removing samples known to be contaminated.			

Supplementary Table 2

Model parameters, intercepts, confidence intervals and significance levels for US dataset. P value tests that the intercept and slopes > 0 using Satterthwaite approximations.

	Estimates	*Standard Error*	*Degrees of Freedom*	*T statistic*	*P-value (>|t|)*
**Fixed Parts**					
(Intercept)	2.02	0.32	530.4	6.28	7.28×10^{-10} (***)
Water table depth (m below land surface)	1.84×10^{-3}	1.02×10^{-3}	2913	1.81	0.071 (.)
Land Elevation (m asl)	-2.66×10^{-4}	5.49×10^{-5}	2915	-4.84	1.34×10^{-6} (***)
Temperature in the Wettest Quarter of the Year (°C)	3.35×10^{-2}	3.49×10^{-3}	2888	9.61	$< 2.00 \times 10^{-16}$ (***)
Temperature in the Warmest Quarter of the Year (°C)	-9.33×10^{-2}	1.09×10^{-2}	2906	-8.53	$< 2.00 \times 10^{-16}$ (***)
Precipitation in the Wettest Month of the Year (mm)	-2.45×10^{-3}	7.51×10^{-4}	2905	-3.27	0.001 (**)
Iron (µg / L)	6.87×10^{-5}	4.97×10^{-6}	2903	13.80	$< 2.00 \times 10^{-16}$ (***)
Dissolved Oxygen (mg / L)	-7.06×10^{-2}	5.53×10^{-3}	2915	-12.80	$< 2.00 \times 10^{-16}$ (***)
Sample Temperature (°C)	4.49×10^{-2}	5.28×10^{-3}	2908	8.50	$< 2.00 \times 10^{-16}$ (***)
pH	-9.61×10^{-2}	2.34×10^{-2}	2907	-4.11	4.06×10^{-5} (***)
Sample Depth (m below ground surface)	-7.77×10^{-3}	6.30×10^{-4}	2899	-12.30	$< 2.00 \times 10^{-16}$ (***)
Precipitation in the Driest Month of the Year (mm)	-9.53×10^{-3}	1.05×10^{-3}	2899	-9.07	$< 2.00 \times 10^{-16}$ (***)
Calcium (mg / L)	4.45×10^{-3}	4.08×10^{-4}	2915	10.90	$< 2.00 \times 10^{-16}$ (***)
Potassium (mg / L)	2.83×10^{-2}	3.82×10^{-3}	2902	7.41	1.69×10^{-13} (***)
Manganese (µg / L)	3.75×10^{-5}	1.81×10^{-5}	2899	2.07	0.039 (*)
Sodium (mg / L)	-5.66×10^{-4}	1.73×10^{-4}	2904	-3.27	0.001 (**)
Silica (mg / L)	-6.29×10^{-3}	1.32×10^{-3}	2914	-4.77	1.97×10^{-6} (***)
Landuse- Natural	-9.74×10^{-3}	4.87×10^{-2}	2911	-0.20	0.841 (.)
Landuse - Urban	0.17	5.31×10^{-2}	2915	3.19	0.001 (**)
**Random Parts**					
σ^2 (1)	0.689				
$\tau_{00,\ Aquifer\ Age}$ (2)	0.215				
$ICC_{Aquifer\ Age}$ (3)	0.238				
Observations	2916				
R^2 / Ω_0^2 (4)	.429 / .429				
AIC (5)	7282.231				
Notes	* p<.05 ** p<.01 *** p<.001 . p>.05<br>(1) Within group variance<br>(2) Between group variance (Aquifer Age)<br>(3) Intraclass correlation coefficient<br>(4) Random slope intercept correlation<br>(5) Akaike information criteria				

Supplementary Table 3

Summary table of annual average temperature and precipitation in the US NWQMC data dataset.

	Annual average temperature (^oC)	Annual average precipitation (mm)
Min.	2.8	94.0
1st Qu.	9.2	480.2
Median	11.5	812.0
Mean	12.6	809.1
3rd Qu.	16.5	1133.0
Max.	24.2	1798.0

Supplementary Table 4

Model coefficient estimates, standard error, t value and p values for simple linear models of log(*DOC concentration (mg / L)*) vs significant individual quantitative variables used in the US model shown for available Australian data. P value is testing that the slope of the linear model log(*DOC concentration (mg / L)*) vs variable is significantly different from 0.

Parameter	Estimate	Std. Error	T value	P value (>|t|)	Change in DOC concentration with one unit increase in parameter for Australian data (US results shown in brackets)
Dissolved Oxygen (mg / L)	-0.05883	0.07011	-0.839	0.40400	-5.713% (-6.812%)
Iron (µg / L)	0.00026	0.00004	6.249	<0.00001	0.026% (0.007%)
Sample Temperature (°C)	-0.22571	0.09605	-2.350	0.02130	-20.205% (4.593%)
pH	-0.39960	0.47140	-0.848	0.39900	-32.941% (-9.162%)
Land Elevation (m above sea level)	-0.00513	0.00131	-3.911	0.00020	-0.512% (-0.027%)
Sample Depth (m below ground surface)	-0.00265	0.00121	-2.183	0.03210	-0.264% (-0.774%)
Temperature in the Wettest Quarter of the Year (°C)	-0.17840	0.03508	-5.085	<0.00001	-16.339% (3.407%)
Temperature in the Warmest Quarter of the Year (°C)	-0.44833	0.09439	-4.750	0.00001	-36.131% (-8.908%)
Precipitation in the Wettest Month of the Year (mm)	0.01606	0.00408	3.938	0.00018	1.619% (-0.245%)
Precipitation in the Driest Month of the Year (mm)	0.04462	0.00887	5.031	<0.00001	4.563% (-0.949%)
Calcium (mg / L)	0.01317	0.00362	3.643	0.00049	1.326% (0.446%)
Potassium (mg / L)	0.18690	0.23410	0.798	0.42700	20.551% (2.869%)
Sodium (mg / L)	-0.00891	0.00447	-1.992	0.04990	-0.887% (-0.057%)
Manganese (µg / L)	0.00015	0.00015	1.052	0.29600	0.015% (0.004%)
Silica (mg / L)	-0.04096	0.01308	-3.131	0.00246	-4.013% (-0.627%)

Supplementary Table 5

Comparison between US and Australian data for climate variables. P-values based on Welch's Two Sample t-test for differences in means for variables with equal variance (temperature in the wettest quarter of the year, precipitation in the driest month of the year and sample temperature) and standard t-test used for variables with equal variance (precipitation in the wettest month). P values test whether the means for each variable are significantly different from 0.

	Temperature in the wettest quarter of the year		Precipitation in the driest month of the year		Precipitation in the wettest month of the year		Sample temperature	
	Australia	United States	Australia	United States	Australia	United States	Australia	United States
Min.	16.2	-0.5	33.0	0.0	72.0	11.0	18.6	4.0
1st Quarter	23.1	18.0	35.0	11.75	93.5	83.0	20.9	12.6
Median	25.3	20.6	35.0	25.0	94.0	107.0	21.6	15.78
Mean	23.2	19.2	42.6	36.3	101.9	104.7	21.57	16.15
3rd Quarter	25.4	23.3	41.0	62.0	97.0	122.0	22.25	19.5
Max.	25.6	27.6	71.0	109.0	165.0	255.0	28.4	34.0
St. Dev.	3.4	6.1	13.5	27.7	31.0	36.0	1.4	4.7
n	79	2916	79	2916	79.0	2916	79	2916
p-value for differences in mean	$< 2.2 \times 10^{-16}$		1.54×10^{-4}		0.507		2.2×10^{-16}	

Supplementary Table 6

Comparison of linear model (log(*DOC concentration (mg / L)*) vs variable) slopes between US and Australian data for climate variables after confining US data to the min and max ranges available in the Australian dataset for the same variables. P values test whether the slope is significantly different from 0.

	Temperature in the wettest quarter of the year		Precipitation in the driest month of the year		Precipitation in the wettest month of the year		Sample temperature	
	Australia	United States	Australia	United States	Australia	United States	Australia	United States
Estimate	-0.178	-0.275	0.045	0.016	0.016	0.007	-0.226	-0.099
Std. Error	0.035	0.041	0.009	0.008	0.004	0.008	0.096	0.045
T value	-5.085	-6.764	5.031	2.014	3.938	0.896	-2.350	-2.200
P value (>|t|)	2.52×10^{-6}	1.483×10^{-9}	3.11×10^{-6}	0.047	1.79×10^{-4}	0.373	0.021	0.030
Adjusted R^2	0.242	0.337	0.238	0.034	0.157	-0.002	0.055	0.042

Supplementary Table 7

Cost of construction, annual operation and household water cost increases resulting from GAC filtration implementation and operation as determined using the US EPA work breakdown structure model for cost estimation of GAC [61]. Calculations assume a system of gravity-fed concrete GAC contactors with an empty bed contact time of 20 minutes, treating a design flow of 6.6 MGD (25 Ml/d).

	$US*	$US/gallon ($US/litre)
Total Capital Cost	4,344,568	0.66 (0.17)
Annual O&M Cost	1,049,545	0.16 (0.04)
Annualized Cost (35.4 years at 7%)	1,384,170	
Annualized cost per 1,000 gallons (3785 litres) average flow	0.57	
Annualized cost per household per year	134	

* Prices at 2014 base date.

Supplementary Notes

Supplementary Note 1

Comparison to Australian groundwater DOC data

The results for each significant ($p < 0.05$) quantitative (chemical and climatic) model variable in the US dataset were compared to simple linear regression models of log(*DOC concentration*) vs. the same variables from the Australian dataset (sample n = 79 after removing any row containing one or more missing value of any variable). The results (Supplementary Table 4) show that the slope directions for all chemical variables match the slope directions in the US model, suggesting that the effect of water chemistry on DOC appears to be globally consistent. The regression analyses also however show that some climate specific variables exhibit an opposite trend in the Australian dataset compared to the US dataset. These include precipitation in the wettest and driest months of the year, temperature in the wettest quarter of the year and sample temperature. Welch's Two Sample t-test for differences in means reveal that the average temperature in the wettest quarter of the year, precipitation in the driest month of the year and sample temperature are significantly higher with lower standard deviations (44%, 51% and 70% lower respectively) in the Australian dataset than the US dataset ($p < 0.001$, Supplementary Table 5 and Supplementary Figure 6). This implies that different climate types have different effects on groundwater DOC concentrations. This was further confirmed by constraining the US dataset to the minimum and maximum ranges available in the Australian dataset for each of the four climate related variables. Simple linear regression models were performed for log(*DOC concentration (mg / L)*) vs. the four individual variables and it was confirmed that the direction of correlation shown by the four simple linear models (Supplementary Table 5) are equivalent to the direction of correlation in the Australian dataset (Supplementary Table 6). Increased temperatures in arid climates such as Australia, limits rather than primes biological activity due to water limitation and low variability in precipitation rates. In these climate types, groundwater DOC is more likely to be sourced by river recharge than diffuse rainfall recharge.

Supplementary Note 2

Calculation of costs associated with implementation of GAC treatment for DOC removal from groundwater

A USA Environment Protection Agency (EPA) costing tool for GAC [61] was used to determine the costs associated with the construction and implementation of GAC (https://www.epa.gov/dwregdev/drinking-water-treatment-technology-unit-cost-models-and-overview-technologies). This was applied to a 25 Megalitre per day (Ml/d) plant (Supplementary Table 7) which was determined to be large enough to achieve economies of scale.

Supplementary References

1. National Water Quality Monitoring Council. Water Quality Data. National Water Quality Monitoring Council (2017).

2. Richts, A., Struckmeier, W.F. & Zaepke, M. WHYMAP and the Groundwater Resources Map of the World 1:25,000,000. In: *Sustaining Groundwater Resources: A Critical Element in the Global Water Crisis* (ed Jones JAA). Springer Netherlands (2011).

3. Wieder, W. Regridded Harmonized World Soil Database v1.2. ORNL Distributed Active Archive Center (2014).

4. Darling, W.G., Sorensen, J.P.R., Newell, A.J., Midgley, J. & Benhamza, M. The age and origin of groundwater in the Great Western Erg sub-basin of the North-Western Sahara aquifer system: Insights from Krechba, central Algeria. *Applied Geochemistry* **96**, 277-286 (2018).

5. Bryan, E., Meredith, K.T., Baker, A., Andersen, M.S. & Post, V.E.A. Carbon dynamics in a Late Quaternary-age coastal limestone aquifer system undergoing saltwater intrusion. *Science of The Total Environment* **607-608**, 771-785 (2017).

6. Gleeson, J., Santos, I.R., Maher, D.T. & Golsby-Smith, L. Groundwater–surface water exchange in a mangrove tidal creek: Evidence from natural geochemical tracers and implications for nutrient budgets. *Marine Chemistry* **156**, 27-37 (2013).

7. Jeffrey, L.C., Santos, I.R., Tait, D.R., Makings, U. & Maher, D.T. Seasonal Drivers of Carbon Dioxide Dynamics in a Hydrologically Modified Subtropical Tidal River and Estuary (Caboolture River, Australia). **123**, 1827-1849 (2018).

8. Jeffrey, L.C., Maher, D.T., Santos, I.R., McMahon, A. & Tait, D.R. Groundwater, Acid and Carbon Dioxide Dynamics Along a Coastal Wetland, Lake and Estuary Continuum. *Estuaries and Coasts* **39**, 1325-1344 (2016).

9. Meredith, K.T., Han, L.F., Cendón, D.I., Crawford, J., Hankin, S., Perterson, M. & Hollins, S.E. Evolution of dissolved inorganic carbon in groundwater recharged by cyclones and groundwater age estimations using the 14C statistical approach. *Geochimica et Cosmochimica Acta* **220**, 483-498 (2018).

10. Meredith, K.T., Cendón, D.I., Hankin, S. & Hollins, S. Allanooka-Casuarinas groundwater dating, Western Australia. Department of Water (2012).

11. Meredith, K.T., Han, L.F., Hollins, S.E., Cendón, D.I., Jacobsen, G.E. & Baker, A. Evolution of chemical and isotopic composition of inorganic carbon in a complex semi-arid zone environment: Consequences for groundwater dating using radiocarbon. *Geochimica et Cosmochimica Acta* **188**, 352-367 (2016).

12. Perkins, A.K., Santos, I.R., Sadat-Noori, M., Gatland, J.R. & Maher, D.T. Groundwater seepage as a driver of CO2 evasion in a coastal lake (Lake Ainsworth, NSW, Australia). *Environmental Earth Sciences* **74**, 779-792 (2015).

13. Sadat-Noori, M., Maher, D.T. & Santos, I.R. Groundwater Discharge as a Source of Dissolved Carbon and Greenhouse Gases in a Subtropical Estuary. *Estuaries and Coasts* **39**, 639-656 (2016).

14. Sadat-Noori, M., Tait, D.R., Maher, D.T., Holloway, C. & Santos, I.R. Greenhouse gases and submarine groundwater discharge in a Sydney Harbour embayment (Australia). *Estuarine, Coastal and Shelf Science* **207**, 499-509 (2018).

15. Santos, I.R., Beck, M., Brumsack, H.-J., Maher, D.T., Dittmar,T., Waska, H. & Schnetger, B. Porewater exchange as a driver of carbon dynamics across a terrestrial-marine transect: Insights from coupled 222Rn and pCO2 observations in the German Wadden Sea. *Marine Chemistry* **171**, 10-20 (2015).

16. Santos, I.R., Cook, P.L.M., Rogers, L., de Weys, J. & Eyre, B.D. The “salt wedge pump”: Convection-driven pore-water exchange as a source of dissolved organic and inorganic carbon and nitrogen to an estuary. **57**, 1415-1426 (2012).

17. Stewart, B.T., Santos, I.R., Tait, D.R., Macklin, P.A. & Maher, D.T. Submarine groundwater discharge and associated fluxes of alkalinity and dissolved carbon into Moreton Bay (Australia) estimated via radium isotopes. *Marine Chemistry* **174**, 1-12 (2015).

18. Andersen, M.S. Fielddata Maules Creek (Elfin) GDE Aug 2007. Unpublished Dataset (2005).

19. Andersen, M.S. Maules Creek (Elfin Crossing Grid File). Unpublished Dataset (2005).

20. Andersen, M.S. Maules Creek Chemistry Oct-Dec 2008. Unpublished Dataset (2005).

21. Andersen, M.S. Maules Creek Fielddata_Feb_2008. Unpublished Dataset (2008).

22. Andersen, M.S. Wellington chemistry lab report 2014. Unpublished Dataset (2014).

23. Andersen, M.S. Wellington field chemistry data 2014. Unpublished Dataset (2014).

24. Andersen, M.S. ICP-2015-221-report. Unpublished Dataset (2015).

25. Andersen, M.S. Anna Bay Chemistry 2014_master sheet. Unpublished Dataset (2015).

26. Andersen, M.S. ICP-2016-161-report. Unpublished Dataset (2016).

27. Andersen, M.S. Wellington field chemistry data 2016_Compiled_Updated May. Unpublished Dataset (2016).

28. Baker, A. ICP-2014-191-report. Unpublished Dataset (2014).

29. Meredith, K.T. Namoi. Unpublished Dataset (2017).

30. Meredith, K.T., Baker, A.B., Andersen, M.S., O’Carroll, D.M., Rutlidge, H., McDonough, L.K., Oudone, P., Bryan, E. & Zainuddin, N. Isotopic and chromatographic fingerprinting

of the sources of dissolved organic carbon in a shallow coastal aquifer. *Hydrol Earth Syst Sci Discuss* **2019**, 1-20 (2019).

31. Santos, I.R. Original Data – Cook Island, Heron Island, Bribie Island. Unpublished Dataset (2018).

32. WaterNSW. Groundwater_DOC. Unpublished Dataset (2017).
33. Water Corporation WA. Perth Timeseries. Unpublished Dataset (2019).

34. Santos, I.R. Brazil and Argentina. Unpublished Dataset (2018).

35. Lapworth, D.J., Zahid, A., Taylor, R.G., Burgess, W.G., Shamsudduha, M., Ahmed, K. M., Mukherjee, A., Gooddy, D.C., Chatterjee, D. & MacDonald, A. M. Security of Deep Groundwater in the Coastal Bengal Basin Revealed by Tracers. *Geophysical Research Letters* **45**, 8241-8252 (2018).

36. Huang, S., Wang, S.Y., Ma, T., Tong, L., Wang, Y., Liu, C. & Zhao, L. Linking groundwater dissolved organic matter to sedimentary organic matter from a fluvio-lacustrine aquifer at Jianghan Plain, China by EEM-PARAFAC and hydrochemical analyses. *Science of The Total Environment* **529**, 131-139 (2015).

37. Zhang, K.P., Zhou, A.G., Zhou, J., Liu, C.F., Cai, H.S., Xu, W., Liu, Y. & Fang, J.J. Characteristics of dissolved organic carbon isotope in groundwater in Shijiazhuang and its environmental implications. *Hydrogeol Eng Geol* **40**, 12-18 (2013).

38. Datry, T., Malard, F. & Gibert, J. Dynamics of solutes and dissolved oxygen in shallow urban groundwater below a stormwater infiltration basin. *Science of The Total Environment* **329**, 215-229 (2004).

39. Artinger, R., Buckau, G., Geyer, S., Fritz, P., Wolf, M. & Kim, J.I. Characterization of groundwater humic substances: influence of sedimentary organic carbon. *Applied Geochemistry* **15**, 97-116 (2000).

40. Chapelle, F.H., Bradley, P.M., Journey, C.A. & McMahon, P.B. Assessing the Relative Bioavailability of DOC in Regional Groundwater Systems. *Groundwater* **51**, 363-372 (2013).

41. Schiff, S.L., Aravena, R., Trumbore, S.E. & Dillon, P.J. Dissolved Organic Carbon Cycling in Forested Watersheds: A Carbon Isotope Approach. *Water Resources Research* **26**, 2949-2957 (1990).

42. Wassenaara, L.I., Aravena, R., Fritz, P. & Barker, J.F. Controls on the transport and carbon isotopic composition of dissolved organic carbon in a shallow groundwater system, Central Ontario, Canada. *Chemical Geology: Isotope Geoscience section* **87**, 39-57 (1991).

43. Gooddy, D. & Hinsby, K. Organic Quality of Groundwaters. In: *Natural Groundwater Quality* (eds Edmunds WM, Shand P) (2009).

44. MacDonald, A. Ethiopia. Unpublished Dataset (2018).

45. MacDonald, A. Iceland. Unpublished Dataset (2018).

46. Sorensen, J.P.R. & MacDonald, A. Kenya. Unpublished Dataset (2018).

47. Lapworth, D.J. & MacDonald, A. Mali. Unpublished Dataset (2018).

48. Lapworth, D.J. Malawi. Unpublished Dataset (2018).

49. Ward, J. & Lapworth, D.J. Malawi. Unpublished Dataset (2018).

50. MacDonald, A. Nepal. Unpublished Dataset (2018).

51. Lapworth, D.J. & MacDonald, A. Nigeria. Unpublished Dataset (2018).

52. MacDonald, A.M., O Dochartaigh, B.E. & Smedley, P.L. Baseline groundwater chemistry in Scotland's aquifers. British Geological Survey (2017).

53. Sorensen, J.P.R. Senegal. Unpublished Dataset (2018).

54. Sorensen, J.P.R. & Lapworth, D.J. Uganda. Unpublished Dataset (2018).

55. MacDonald, A. Uganda (2). Unpublished Dataset (2018).

56. Baker, M.A., Valett, H.M. & Dahm, C.N. Organic carbon supply and metabolism in a shallow groundwater ecosystem. *Ecology* **81**, 3133-3148 (2000).

57. Leenheer, J.A., Malcolm, R.L., McKinley, P.W. & Eccles, L.A. Occurrence of dissolved organic carbon in selected ground-water samples in the United States. *Journal of Research of the US Geological Survey* **2**, 361-369 (1974).

58. Santos, I.R., Burnett, W.C., Dittmar, T., Suryaputra, I.G.N.A. & Chanton, J. Tidal pumping drives nutrient and dissolved organic matter dynamics in a Gulf of Mexico subterranean estuary. *Geochimica et Cosmochimica Acta* **73**, 1325-1339 (2009).

59. Luzius, C., Guillemette, F., Podgorski, D.C., Kellerman, A.M. & Spencer, R.G.M. Drivers of Dissolved Organic Matter in the Vent and Major Conduits of the World's Largest Freshwater Spring. *Journal of Geophysical Research: Biogeosciences* **123**, 2775-2790 (2018).

60. Sorensen, J.P., Lapworth, D.J., Marchant, B.P., Nkhuwa, D.C., Pedley, S., Stuart, M.E., Bell, R.A., Chirwa, M., Kabika, J., Liemisa, M. & Chibesa, M. In-situ tryptophan-like fluorescence: A real-time indicator of faecal contamination in drinking water supplies. *Water Research* **81**, 38-46 (2015).

61. US EPA. Drinking Water Treatment Technology Unit Cost Models and Overview of Technologies (2017).
